# A Unique Presentation of 3-Methylcrotonyl-CoA Carboxylase Deficiency

**DOI:** 10.7759/cureus.39401

**Published:** 2023-05-23

**Authors:** Ashwin Jagadish, Kaitlin Sclater, Taylor Lapinski, Karen Adkins, Lauren Selzer

**Affiliations:** 1 Pediatrics, East Tennessee State University James H. Quillen College of Medicine, Johnson City, USA

**Keywords:** leucine, newborn screening, pediatric genetics, pediatrics, 3-methylcrotonyl-coa carboxylase deficiency

## Abstract

3-methylcrotonyl-CoA carboxylase deficiency is an autosomal recessive disorder resulting in impaired leucine metabolism. The condition is typically diagnosed with newborn screening; patients diagnosed at a later stage generally present with symptoms including metabolic disturbances, seizures, failure to thrive, or delayed development. We present the case of a child diagnosed at 12 months of age who was noted to have recurrent viral infections and nonspecific gastrointestinal symptoms of vomiting, hematochezia, and gaseous distention of the abdomen. Newborn screening did not reveal any abnormalities. Evaluation for underlying immunodeficiency was unremarkable; genetic testing revealed bi-allelic mutations in MCCC2, a known association of 3-methylcrotonyl-CoA carboxylase deficiency. It is important to consider genetic disorders when evaluating patients even if the newborn screening is unremarkable.

## Introduction

The autosomal recessive disorder known as 3-methylcrotonyl-CoA carboxylase (3MCC) deficiency involves mutations in the MCCC1 or MCCC2 genes and impaired leucine metabolism [1]. The condition’s prevalence is between 1:2,400 and 1:68,000 based on the population being studied and is commonly diagnosed via newborn screening [2]. Presentation of this condition can vary from substantial neurologic dysfunction to adults who are asymptomatic [3]. Children diagnosed after the newborn period can present with seizures, delayed development, failure to thrive, or metabolic abnormalities [2]. There is no clear connection between genotype and phenotype in terms of clinical presentation of this condition [4].

## Case presentation

A 2,935 g full-term female was born via spontaneous vaginal delivery to a gravida 1 para 1 female. The child’s parents were in a non-consanguineous relationship. During the pregnancy, the child’s mother tested positive for Group B Streptococcus, for which she received intrapartum antibiotics as prophylaxis. Prenatal vitamins were taken throughout the pregnancy. The child’s mother had a history of herpes simplex virus treated with famciclovir suppression therapy, Ehlers-Danlos type III, anxiety, and depression.

**Figure 1:**
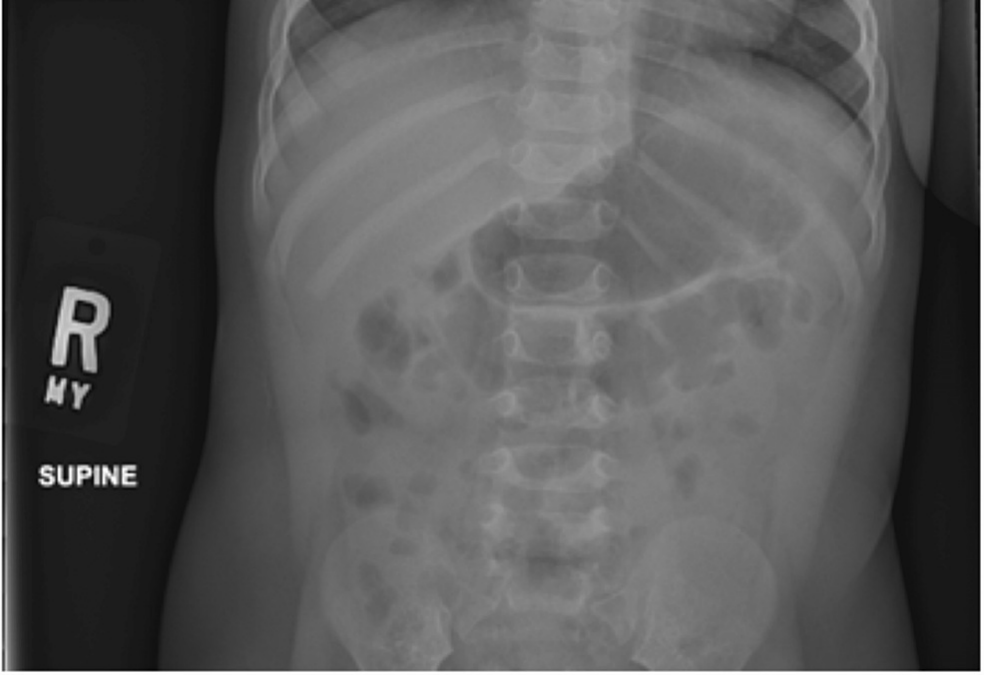
X-ray of the abdomen - first view

**Figure 2:**
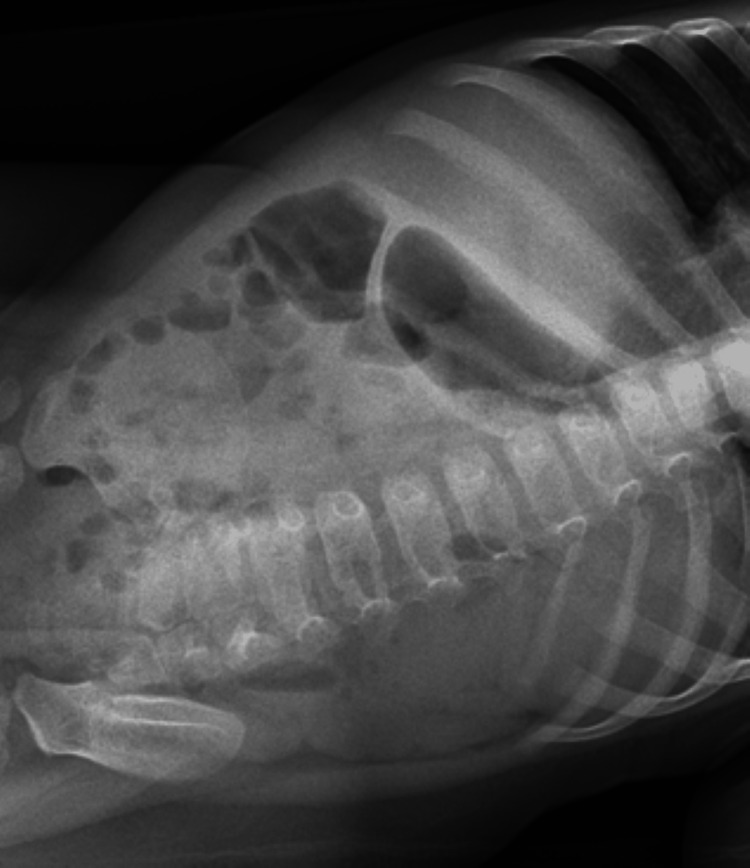
X-ray of the abdomen - second view

At the age of two months, the child was admitted to the hospital for respiratory syncytial virus accompanied by episodes of vomiting. Three months later, she presented to the emergency department, where she was diagnosed with coronavirus OC43. Two months later, at the age of seven months, she was admitted for constipation and dehydration. During this admission, her abdominal x-ray did not reveal any acute findings. The following month, she was admitted twice for vomiting and bloody stools. At that time, her x-ray findings revealed non-obstructive bowel gas patterns and gaseous distention of the colon (Figures 1, 2). Three months later, she was referred to a genetics specialist for further evaluation.

Laboratory results for Immunoglobulins A, G, and M were within normal limits. Genetic testing revealed bi-alellic mutations in MCCC2, which are associated with 3MCC deficiency. The acylcarnitine panel was unremarkable other than an OH-Isovalerylcarnitine, C5OH level of 0.05 nmol/mL (normal range ≤ 0.04 nmol/mL). Testing for total carnitine, free carnitine, homocysteine, ammonia, lipase, amylase, lactic acid, and vanillylmandelic acid resulted in values within normal limits. Urine organic acids testing was ordered but not completed by the patient. Newborn screening results were reviewed and confirmed to be normal (Table 1). The child’s parents were advised to limit her protein consumption. Additionally, supplementation with carnitine would be considered based on disease progression.

**Table 1:** Newborn Screening Results

Disorder/Profile	Result	Remarks	Normal Values
Congenital Hypothyroidism	Within Normal Limits	Normal	< 32 µU/mL
Galactosemia	Within Normal Limits	Normal	GAL < 13 mg/dL GALT ≥ 3.48 U/dL
Hemoglobinopathies	FA	No Hemoglobinopathies Observed	FA, AF for Older Infants
Biotinidase Deficiencies	Within Normal Limits	Normal	≥ 44.64 U/dL
Congenital Adrenal Hyperplasia	Within Normal Limits	Normal	< 37 ng/mL
Amino Acid Profile	Within Normal Limits	Normal	Within Normal Limits
Fatty Acid Profile	Within Normal Limits	Normal	Within Normal Limits
Organic Acid Profile	Within Normal Limits	Normal	Within Normal Limits
Cystic Fibrosis	Within Normal Limits	Normal	< 54 ng/mL
Lysosomal Disorders	Within Normal Limits	Normal	Within Normal Limits
X-linked Adrenoleukodystrophy	Within Normal Limits	Normal	Within Normal Limits
Severe Combined Immunodeficiency	Within Normal Limits	Normal	Within Normal Limits
Spinal Muscular Atrophy	Within Normal Limits	Normal	Within Normal Limits

Four months later, she presented to the hospital twice. The first visit was to the emergency department for human rhinovirus/enterorivus. The second visit, three weeks later, involved admission for human herpes virus 6 encephalitis. The following month, she presented to the emergency department for human rhinovirus/enterovirus. Five months later, she was admitted for human rhinovirus/enterovirus. One month later, the patient was admitted for gastroenteritis, human rhinovirus/enterovirus, and adenovirus.

## Discussion

The patient described in this case was born at term and had normal newborn screening results. Soon after birth, she began to have repeated viral infections including coronavirus OC43, multiple episodes of human rhinovirus/enterovirus, HHV6 encephalitis, gastroenteritis, and adenovirus. The frequency of her infections was concerning. As a result, she was referred to a genetics specialist for further evaluation. Genetic testing revealed bi-alellic mutations in MCCC2, which is known to be associated with 3MCC deficiency. The remained of her labs were non-contributory.

Individuals with 3MCC deficiency require special diets that are high in calorie content but in which high-protein foods are limited [5]. Suitable consumption of leucine is considered 35-65 mg/kg birth weight for individuals between the age of four and seven [5]. Extra consumption of essential and non-essential amino acids, other than leucine, is needed to ensure appropriate growth and development [5].

In patients with 3MCC deficiency, free carnitine levels in the plasma are indicative of free carnitine levels within the muscle [6]. Muscle is the primary repository of carnitine within the human body [6]. Supplementation with L-carnitine is known to increase plasma levels of carnitine [6]. There is inconclusive evidence on the risks and benefits of supplementation with L-carnitine [6]. Recommendations include no indication for supplementation, supplementation when levels are low, and always supplement [6]. The long-term consequences of carnitine are not well studied [6].

Newborn screening includes education of parents, screening of the infant, disorder evaluation and management, and following-up as needed [7]. About 3,400 infants receive intervention early on due to the identification of disorders on newborn screening tests [7]. In the United States, the Recommended Uniform Screening Panel includes 35 main diseases and 26 secondary diseases, one of which is 3MCC deficiency [7]. Given that our patient did not test positive for the disorder on newborn screening, we recommend consideration of genetic disorders, especially 3MCC deficiency, in young children with unexplained repetitive illnesses.

## Conclusions

Patients with 3MCC deficiency can present with a wide variety of symptoms. The patient in this case had repeated viral infections requiring frequent visits to the emergency department. In addition, many of these visits resulted in inpatient admission. There was a concern for underlying genetic causes, so she was referred to a genetics specialist for further evaluation. Genetic testing indicated bi-allelic mutations MCCC2, which are associated with 3MCC deficiency. This condition is often diagnosed with newborn screening, but cases such as the one we present can escape detection. It is important to consider genetic disorders as a cause of repetitive illness in patients, even if newborn screening does not detect abnormalities.
